# Federated Analysis for Privacy-Preserving Data Sharing: A Technical and Legal Primer

**DOI:** 10.1146/annurev-genom-110122-084756

**Published:** 2023-05-30

**Authors:** James Casaletto, Alexander Bernier, Robyn McDougall, Melissa S. Cline

**Affiliations:** 1 Genomics Institute, University of California, Santa Cruz, California, USA;; 2 Centre of Genomics and Policy, Faculty of Medicine and Health Sciences, McGill University, Montreal, Quebec, Canada;

**Keywords:** federation, federated analysis, privacy protection, GDPR, genomics

## Abstract

Continued advances in precision medicine rely on the widespread sharing of data that relate human genetic variation to disease. However, data sharing is severely limited by legal, regulatory, and ethical restrictions that safeguard patient privacy. Federated analysis addresses this problem by transferring the code to the data—providing the technical and legal capability to analyze the data within their secure home environment rather than transferring the data to another institution for analysis. This allows researchers to gain new insights from data that cannot be moved, while respecting patient privacy and the data stewards’ legal obligations. Because federated analysis is a technical solution to the legal challenges inherent in data sharing, the technology and policy implications must be evaluated together. Here, we summarize the technical approaches to federated analysis and provide a legal analysis of their policy implications.

## INTRODUCTION

Most diseases have a genetic component (49). While clinical genetic testing now offers individuals greater opportunities to understand and manage their heritable disease risk (39, 79), its impact is limited by the many gaps in our understanding of human genetic variation. This is seen in the significant missing heritability of most diseases, with family history predicting disease risk more accurately than genetics (57, 91). It is also seen in the high rates of variant of uncertain significance (VUS) results in clinical testing, with recent studies reporting VUS results in approximately 20% of the patients tested with cancer susceptibility gene panel tests (21, 40, 72). Patients of non-European ethnicities have a significantly higher rate of VUS results than their European counterparts, leading to greater mortality from heritable disorders (14, 68). These problems could be addressed by data sharing, particularly global data sharing. However, the sharing of human genetic data is limited by a complex network of legal, ethical, and regulatory restrictions that aim to protect patient privacy (6) or data sovereignty (44, 54, 71, 83). As a result, most human genetic data remain siloed and are inaccessible to most researchers and those making clinical inferences.

Often, however, these regulations permit the sharing of aggregated, nonidentifiable data, which can help advance research. For example, variant interpretation guidelines from the American College of Medical Genetics and Genomics and the Association for Molecular Pathology (67) include summary statistics that describe the enrichment or lack of disease in patients with a given genetic variant but do not directly require individual observations of those patients. If one can share the capabilities to generate these summary statistics, one can share knowledge without transferring the sensitive patient data.

**Figure 1:**
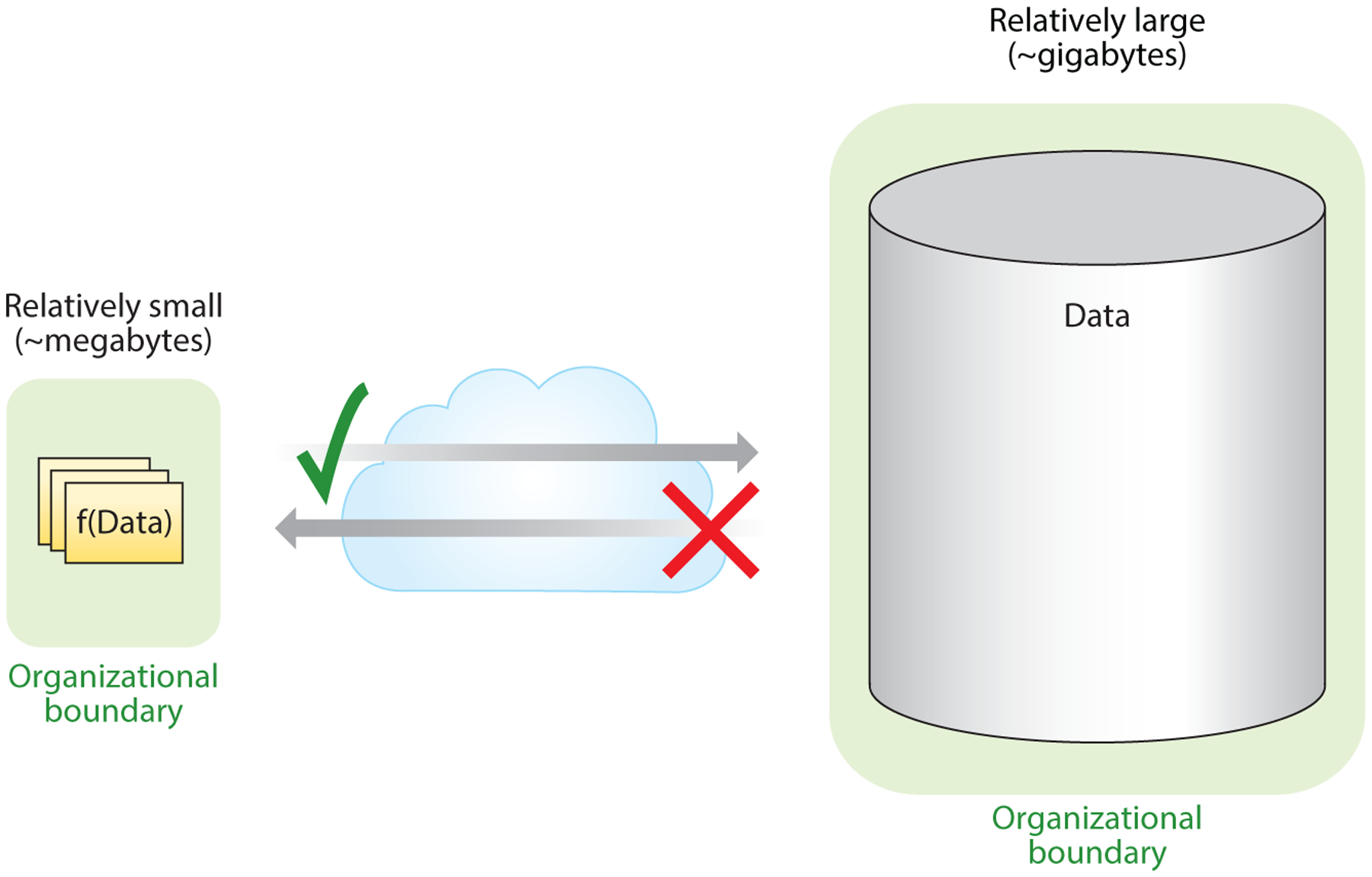
Illustrating the logic of “bringing the code to the data,” either because the data are much larger relative to the analytical code [f(Data)] or because of restrictions on exporting the data across organizational boundaries.

Data federation achieves such sharing through a decentralized architecture, in which a network of data providers maintains full control over the data within a secure computing environment while enabling access to the data by external collaborators (81). Federated analysis is a form of data federation in which collaborators “bring the code to the data” to analyze data in situ, within the data providers’ computing environment (see Figure 1).

When federated analysis generates nonidentifiable results from patient-level data, those results can be shared externally even when the original patient-level data cannot. Federated analysis balances the needs of data stewards to restrict access to regulated data with the needs of the scientific and clinical community to gain new insights from data that cannot be transferred for legal, ethical, or technical reasons. Achieving this vision requires technologies that allow researchers to reliably analyze data that they cannot directly access, coupled with privacy safeguards that allow data stewards to assess the risks inherent in such analyses. In short, federated analysis offers technical solutions to legal restrictions on data use and data sharing. As such, to understand its potential and limitations, one must consider the technical and legal aspects of the situation together.

This article begins with a discussion of privacy in genomics and how that privacy can be compromised. Next, we discuss the central concepts underpinning the General Data Protection Regulation (GDPR), the principal legal framework that regulates the use of personal data in the European Union and European Economic Area. The GDPR has been selected instead of other national and international data protection and privacy norms because it is among the strictest and most influential data protection laws in the world. The conclusions of the article are nonetheless generalizable to ensure compliance with most other national data protection norms, such as the Health Insurance Portability and Accountability Act (HIPAA) in the United States. We next summarize approaches to federated analysis, presenting examples of successful applications and evaluating both the technical approaches and legal implications. Finally, we review areas for further progress on both the technical and legal fronts, presenting overall recommendations for the technical and organizational implementation of federated data analysis.

## PRIVACY AND PRIVACY ATTACKS

The sequencing of the human genome enabled unprecedented opportunities for the biomedical research community to make discoveries that are actionable in human health and precision medicine. It used to be that preserving the privacy of participants in biomedical research equated to maintaining the confidentiality of personally identifiable information and protected health information by either publishing aggregated data or removing these types of data. However, over the years, researchers have found ways to uncover protected information from such datasets. In 2008, the Wellcome Trust and the National Institutes of Health removed access to genomic datasets after it was shown that people could be reidentified from data aggregated from genome-wide association study (GWAS) experiments. For some organizations, removing personally identifiable information and protected health information or publishing only aggregate data was not sufficient to protect the privacy of research participants (38).

### Privacy Threat Model

A privacy threat model defines the most probable attacks on private data, the actors perpetrating the attack, and how the actors would carry out the attack. The actors in a privacy threat model are the entities (people, groups, or organizations) that have some form of access to the data. It is impossible to protect against every attack, so it is necessary to focus on either the most likely or the most detrimental attacks.

In a federated environment where the upstream data contributors, the data custodians, and the downstream data users belong to different organizations, the threat model becomes more complex because the system could be threatened by participants in any of these organizations. One cannot assume that all participants are 100% reliable, but one might assume that some of the participants are reliable, as otherwise there would be no incentive to participate in the system. In short, the safest assumption is that some entity within the system is a potential threat.

A few privacy threat models have been developed and proposed, including the Cloud Security Alliance (CSA), the European Network Information and Security Agency (ENISA), and Link-ability, Identifiability, Non-Repudiation, Detectability, Disclosure of Information, Unawareness, and Non-Compliance (LINDDUN) (96). Whereas the CSA does not provide specific details for how to preserve privacy, ENISA and LINDDUN, while comprehensive, require a significant investment in time and training to understand and incorporate into a secure, privacy-preserving framework. More purpose-designed approaches, such as cloud privacy threat modeling, may be better suited for designing, implementing, and deploying federated solutions when time and resources are not abundant (34).

### Cyber Attacks

Cyber attacks are attempts to read, modify, or delete information through unauthorized access to computer systems. They put the integrity or availability of an otherwise trusted system at risk. For example, a denial-of-service attack is one in which the attacker runs a workload against a service that renders some or all the service either compromised or unavailable. Man-in-the-middle attacks involve an unknown third party that can intercept network communications between two otherwise honest entities and impersonate one or both of them. One of the most common and severe cyber attacks is Structured Query Language (SQL) injection, in which malicious or malformed SQL code is inserted in a web form that is subsequently unwittingly processed by a SQL service on the back end of the web form. Such attacks can obtain unrestricted access to databases with sensitive information, resulting in identity theft, loss of information, and fraud (37).

In the context of federated computing, cyber attacks can be largely mitigated by following commonsense IT security protocols. Federations should require all members to belong to the same virtual private network to block outside traffic. They should use public key cryptography and sufficient authorization requirements within the federation to prevent any leakage of personally identifiable information or protected health data between federation members. And they should leverage other security measures, such as firewalls and multifactor user authentication, across the federation and on a per-host basis.

There are more subtle forms of attack, however, that can be levied within federated environments or in the models that are built and published from federated environments, as described in the following sections.

### Reidentification Attacks

Some privacy attacks require some external data source that contains overlapping data or metadata such that the datasets may be joined to provide a more comprehensive description of an entity such as a person. The ability to join data from multiple sets on some common identifier is called linkability, and the attacks that exploit it are called linkage attacks. Such an external data source might not be available today but might become available in the future. A reidentification attack occurs when data that have been anonymized or pseudonymized become personally identifiable, an example of which is the 1997 reidentification of Massachusetts governor William Weld (80). This reidentification attack required having full ZIP codes, complete birth dates, and genders specified in both health plan data and voter registration data; this linkage enabled the connection between Governor Weld’s identity in the voter registration data and his medical records in the health plan data. This attack could be prevented by masking ZIP codes to two digits and using only birth years, for example. If those covariate data do not contribute to the utility of the shared dataset, then they should be omitted entirely. This illustrates the principle of data minimization, a fundamental principle of computational data privacy (73).

### Reconstruction Attacks

A reconstruction attack is the ability to partially or entirely reconstruct private data from published aggregate data. In this scenario, a trained model or aggregated dataset is produced from data that contain potentially sensitive information and then shared; subsequently, attackers attempt to infer or reconstruct the sensitive information. This broad form of attack includes membership inference and property inference.

A membership inference attack exploits the ability to determine whether a person comes from a source dataset. Methods by which membership attacks may be leveraged were well documented by Shokri et al. (77). One example of membership inference is the attack proposed by Homer et al. (42), in which they demonstrated that it is possible to determine whether a person’s genomic data were used in the creation of published statistics in a GWAS. Given knowledge of the allele frequencies in the population, the allele frequencies in the GWAS mixture, and the genotype information of the person of interest, the attacker uses the allele frequencies to calculate how far the person of interest is from the reference population and the GWAS mixture; the further away the person is, the higher the confidence in the membership inference becomes. In another example (78), researchers demonstrated that an attacker who possesses a person’s genomic sequence can determine that person’s membership in a Beacon, including Beacons that relate to disease, and in this way the Beacon network could leak some of that person’s protected health information.

A property inference attack attempts to infer some aggregate information about the training set as a whole, such as the environment where the data were produced or the percentage of the data that comes from a particular class (i.e., exploiting skewness) (2, 32). It requires that the attacker have auxiliary datasets that contain some property of interest. With these auxiliary datasets, the attacker can build shadow models for each property of interest and then create a classifier that compares results from the target model against these shadow models to distinguish whether the property in question belongs to the target model. For example, Humbert et al. (45) demonstrated that certain single-nucleotide polymorphisms (SNPs) of family members related to Henrietta Lacks could be inferred using available genomic data, family relationship structure, rules of Mendelian inheritance, minor allele frequencies of the SNPs, and linkage disequilibrium among the SNPs.

### Model-Poisoning Attacks

In contrast to the types of attacks discussed above, model-poisoning attacks may occur within a federation while a model is being built or analysis is being performed on private data. Possible objectives include (*a*) a denial-of-service attack that simply renders the model ineffective for predictions using out-of-distribution data and (*b*) label flipping, which targets a subpopulation of the training data such that model predictions involving that subpopulation are erroneous. Yet another, more sophisticated objective of model poisoning is using that information after the model is built to make inferences about the dataset (e.g., property or membership inference attacks) (47).

The two types of model-poisoning attacks involve either data misconduct or model misconduct (51). Model misconduct involves changing how the analysis is performed to alter the outcome, while data misconduct requires that the adversary insert data sufficient to alter the model predictions. For example, if a model that classifies images is trained using images available on the internet, then an attacker can poison that model by uploading poisoned images to the internet. The ways to mitigate this risk include limiting the contribution of any single entity, analyzing the nature of the updates to the global model on a per-contributor basis, and performing outlier detection after the model is built. However, models can be poisoned unintentionally as well. For example, the unintentional underrepresentation of non-European populations in GWASs arguably poisons GWAS models against these underrepresented populations (63).

## INTRODUCING THE GENERAL DATA PROTECTION REGULATION

The GDPR regulates the use of identifiable personal data: data relating to a person who is identified or identifiable. For data to be considered identifiable, there must be a “means likely reasonably to be used either by the controller or by any other person to identify the [concerned individual]” (10, p. 2). This is not the case if reidentification is “practically impossible on account of the fact that it requires a disproportionate effort in terms of time, cost and man-power, so that the risk of identification appears in reality to be insignificant” (10, p. 9). If the data controller and proximate third parties do not have a mechanism enabling the reidentification of the concerned individual that is “likely reasonably to be used,” then the data are considered to be anonymized and therefore not regulated by the GDPR (9).

There is an apparent tension between the manner in which the Court of Justice of the European Union characterizes this legal test (10) and the manner in which it is articulated in the text of the GDPR (28). The GDPR calls for a contextual assessment of the reasonable likelihood of reidentification, which suggests that in circumstances in which reidentification is possible but either improbable or impracticable, the data should be considered nonidentifiable and therefore not regulated (28). The Court of Justice of the European Union, on the other hand, appears to characterize data as identifiable unless reidentification is nearly impossible (10). Nonetheless, both appear to confirm a contextual, risk-based approach to the evaluation of data identifiability (30).

The more restrained reading of the GDPR identifiability criteria should be preferred, as this interpretation limits the application of the GDPR’s onerous procedural requirements to information that poses a material risk of causing individual reidentification, assessed from the perspective of the data controller. If too many data are considered regulated personal data despite posing a limited risk of reidentification, this could frustrate the functioning of data protection legislation. Furthermore, if identifiability is assessed not from the perspective of the data controller and data processor but from the perspective of all third parties, it becomes difficult or impossible for regulated parties to determine the boundaries of their legal responsibilities (10, 28, 64).

The broad framing of identifiable personal data is a potentially unfortunate public policy choice. GDPR-regulated entities have limited financial, human, and technical resources for ensuring their compliance with the data protection regulation. If data identifiability standards are framed to capture a broad range of data that have a limited risk of reidentification, this framing could prompt regulated actors to scale down their data-sharing activities due to the high burden of regulation. It also encourages regulated actors to direct their limited compliance resources to the majority of the data that these actors process rather than structuring their compliance activities in a risk-adjusted manner (i.e., directing their legal compliance resources at the data that have the highest chance of being reidentified). This could lead to actors performing subpar data protection compliance because they lack sufficient resources to ensure appropriate compliance. It could also lead to actors reducing their data sharing due to the potential for GDPR noncompliance, even in circumstances where the data sharing offers large benefits to society or the individual and poses privacy risks that are limited or nonexistent (7).

If data are identifiable, one must consider the role and the associated legal responsibilities that the GDPR ascribes to the actors that use data or that determine how data are used. The GDPR uses the concepts of controller, joint controller, and processor to determine the legal obligations of an actor who uses identifiable personal data. Each of these roles bears distinct legal responsibilities. The determination of whether an actor is a controller, joint controller, or processor is left to the supervisory authorities (i.e., national regulators) and courts. This means that these roles and responsibilities are determined not by the actor’s choice but rather by the manner in which the actor uses data.

The GDPR defines data processing as “any operation or set of operations which is performed on personal data or on sets of personal data, whether or not by automated means” (28, article 3). It goes on to enumerate a nonexhaustive list of examples. In essence, all actions that entail the use or storage of identifiable personal data fall within the definition of data processing (28).

Controllers determine the “purposes and means” of personal data processing (28, article 3). Processors perform personal data-processing activities at the instruction of controllers. In short, data controllers decide what will be done with personal data, while processors implement these decisions. Accordingly, the data controller bears greater legal responsibilities than the data processor (28).

**Figure 2:**
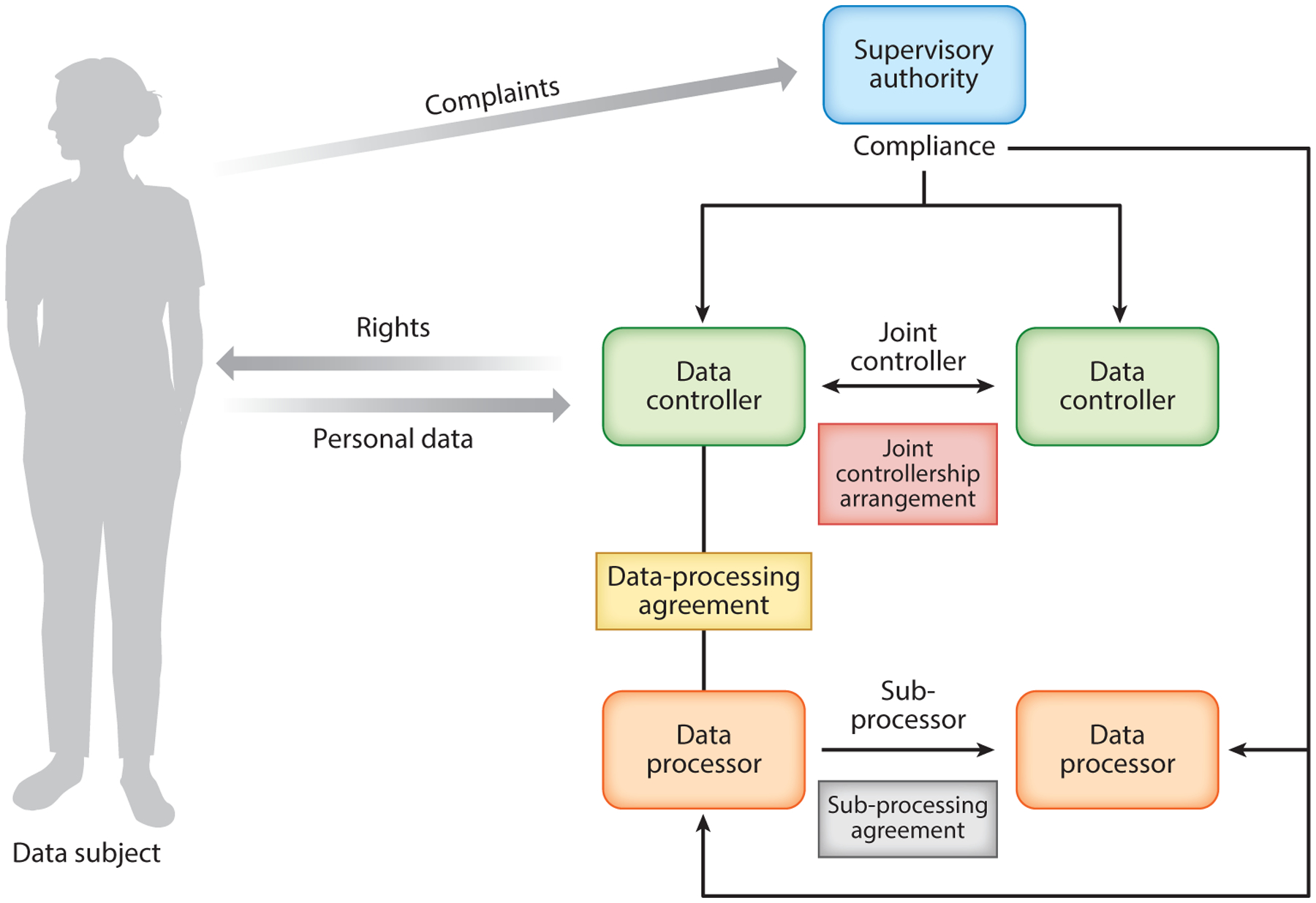
The General Data Protection Regulation: roles and responsibilities.

In some instances, multiple actors will collaborate in determining the purposes and means of personal data processing. This could be the case, for example, if a central organization coordinates and determines the conditions according to which third parties will collect and use personal data for their own purposes (27, 28). In such instances, the law would categorize these multiple actors as joint controllers (28). The GDPR requires joint controllers to establish their respective and overall responsibilities among themselves, using contracts or another form of arrangement. If the collective joint controllers are held liable for some data breach, then each joint controller can be held fully responsible for harms that arise from the action of other controllers in the network. The sole ground that enables any single controller to not be held liable for the actions of the others is for this controller to “[prove] that it is not in any way responsible for the event giving rise to the damage” (28, article 82). Figure 2 shows a graphical depiction of legal entities and their relationships in GDPR.

## DATA PROTECTION IMPLICATIONS OF FEDERATED COMPUTING

Providing guidance on compliance with the substance of the GDPR lies outside the ambit of this article. Rather, we aim to aid health sector data stewards in determining how the structure of their federated data analysis networks, from both a technical and an organizational standpoint, determines the characterization of their activities according to the GDPR—i.e., whether the GDPR would understand them to be controllers, processors, or neither. The characterization first considers whether the concerned actor is engaged in the processing of identifiable personal data, and then considers whether this actor could be characterized as a controller (bearing more responsibilities) or a processor (bearing fewer responsibilities).

A broader public policy context animates this analysis. The GDPR has been strongly criticized as an impediment to the research use of data, the advancement of precision medicine, and the functioning of the healthcare system in general (36). Several arguments support this position. Inherent ambiguities in the language of the GDPR create difficulties in determining how to ensure compliance, even when actors behave in good faith (59). Determinations regarding the appropriate use of data, which are traditionally left to health sector data stewards and research ethics committees, are shifted to generalist data protection regulators, who do not necessarily possess the specialized, domain-specific knowledge required to apply the standards of the GDPR to the health sector (89). Procedural requirements, such as maintaining data-processing reports and documenting self-assessments, can overwhelm the limited resources available to many institutions (58). Finally, the consequences of noncompliance, which can include poor public perception, administrative fines, and civil liability, can deter health sector institutions from exploring data-sharing activities (31). This perverse incentive is especially strong when one institution’s participation would greatly benefit the larger network, but participating in the network would not benefit that institution as greatly (31).

Bearing in mind the foregoing critiques of the GDPR, we can identify the critical roles in a federated data analysis network as follows (81): (*a*) nodes that are responsible for collecting data from individuals and for contributing such data to the network; (*b*) nodes that act as technical data stewards by providing the infrastructure that supports the data storage and processing; (*c*) nodes that act as institutional data stewards, determining which actors can participate in the network as upstream contributors or as downstream recipients of analysis results (and the conditions of such participation); and (*d*) nodes that act as downstream recipients of analysis results and can submit analysis queries to the network and receive responses (81). Since each node that must perform GDPR compliance activities bears compliance costs in entering the network, the burden of compliance activities scales in proportion to the number of nodes in the network (81).

One principal advantage of federated data analysis is to enable scalable access to larger quantities of data. There is a tension between the scalable nature of the federated analysis network from a technical perspective and the inexorable growth of activities required to ensure the network’s compliance. That is, certain legal compliance activities, such as performing data protection impact assessments, retaining records of data-processing activities, or ensuring the alignment of data-processing activities with the principles of the GDPR, can require intensive and repetitious human effort. This can lead to circumstances in which data processing is cost-effective and scalable, but establishing records of compliance with select formalistic, procedural elements of the GDPR is prohibitively cost-intensive (28). It is advantageous to structure networks to reduce the number of regulated data controllers and data processors, in order to enable more streamlined compliance and ensure that the network remains open to a broad range of prospective nodes, including those that lack the significant resources required to perform burdensome regulatory compliance activities.

In short, nodes in a federated data analysis network should use technical and organizational measures to ensure that the benefits of data analysis are maximized without most network nodes engaging in personal data processing, whether as controllers or processors (41). If the data analysis is structured such that most participating nodes do not process potentially identifiable data, using both organizational and technical safeguards, then these nodes will not be required to engage in GDPR compliance activities, and the other nodes are not required to consider these nodes as part of their own GDPR compliance efforts. This ensures that the compliance of the network is cost-effective and simple.

## DATA FEDERATION AND PRIVACY MECHANISMS

Most applications run on a single system. A distributed application is written to run across more than one system to leverage the compute, memory, and/or storage resources of multiple systems. For such applications, the programmer must be provided an abstraction that ignores the physical location of the data (52). Federated computing is a form of distributed computing wherein some or all of these data are subject to the stewardship of an entity other than that which provides and/or runs the application. That is, the analytical software is transferred to the location where the data reside rather than the data being exported for analysis. This approach is particularly well suited for datasets that are either too large or too sensitive to move between organizations (15, 81).

**Figure 3:**
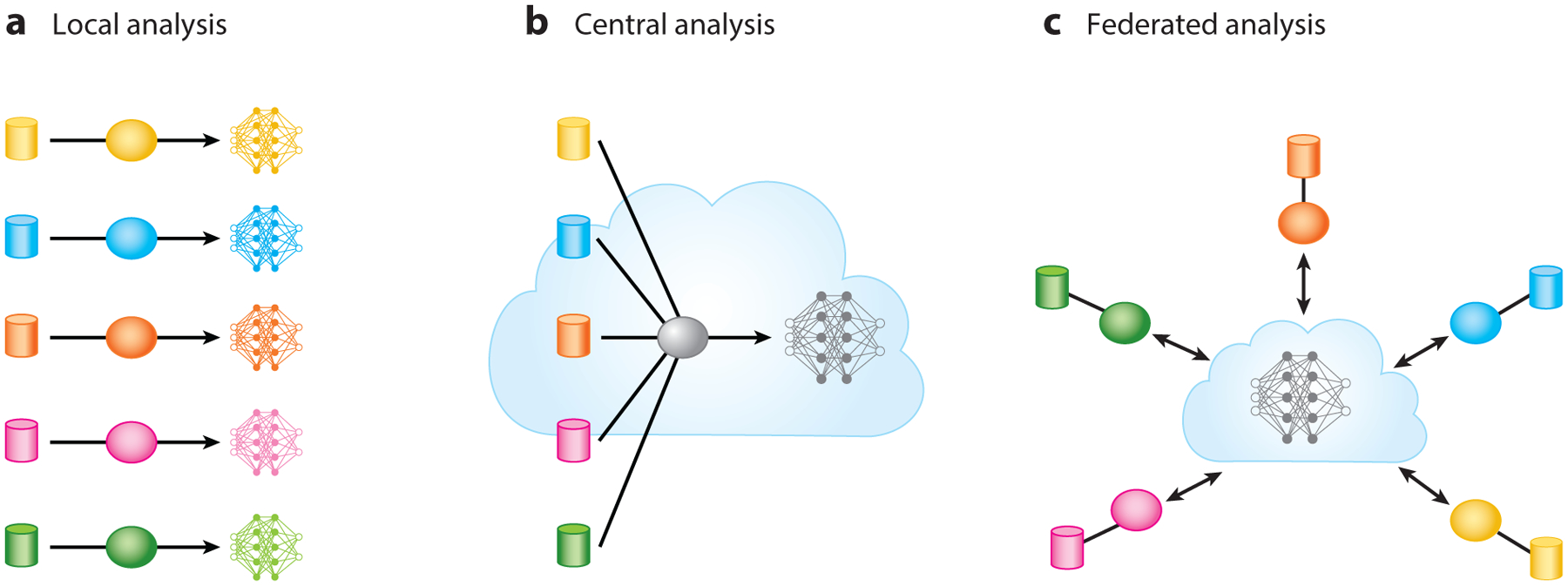
Different ways in which data are analyzed to build models and databases. (*a*) Siloed datasets used for local analysis, where models and databases do not contain other datasets. (*b*) Centralized datasets exported from multiple organizations to a single place where models and databases are built. (*c*) Data federated from multiple institutions to build a single model or database.

There are many forms of federated computing. In federated learning, for example, models are trained over remote datasets in siloed data centers, personal computers, cell phones, and other edge devices while keeping data localized (53). A federated database, by contrast, is a collection of databases that operate as if they were a single database from a unified portal (76). The Gene Expression Omnibus is a federated database of microarray, next-generation sequencing, and other high-throughput functional genomics data (18). Federated analytics, yet another form of federated computing, distributes predictive or descriptive analytic tasks over one or more systems (92). In this article, we focus on descriptive analytics using genomic data. Figure 3 depicts the different ways in which data are analyzed based on their physical location.

All federated methods involve cooperation between people and organizations and sharing some form of potentially sensitive information. In this section, we discuss different privacy mechanisms that may be used within a federation to eliminate any privacy leakage between federation members.

### Secure Multiparty Computation

Secure multiparty computation (SMC), also known simply as multiparty computation, was originally formulated as a research question called the millionaire’s problem, in which two or more people are interested in knowing which of them is richer without revealing their actual wealth and without the help of a trusted third party (60, 97). The foundation of SMC entails secret sharing that leverages zero-knowledge proofs, techniques that enable a prover to prove a claim to one or more verifiers in such a way that they are convinced of its truth without the prover revealing the assertion or any party witnessing the interaction (35). SMC protocols have a correctness requirement that guarantees that either the output is correct or the protocol terminates early. The number of adversaries (*t*) that the protocol can tolerate and still be correct (i.e., either produce correct output or terminate the protocol) depends on the type of secret sharing. Using additive secret sharing, the protocol can tolerate all but one honest participant (*t* < *n*). Using Shamir’s secret sharing, the protocol can tolerate up to *t* < *n*/2 passive adversaries and up to *t* < *n*/3 active adversaries. Examples of SMC in GWAS analyses include work by Constable et al. (19), presented at the 2015 iDASH Privacy and Security Workshop, and by Cho et al. (17), which used data from the Database of Genotypes and Phenotypes (dbGaP).

SMC is an ideal protocol to leverage in a genomics federation. It is purpose built for minimizing privacy leakage while maximizing utility among multiple participants operating on local data to construct global results.

### Homomorphic Encryption

Encryption is the process of encoding data in such a way that they cannot be interpreted without decoding. The stronger the encryption is, the less likely it is that the data can be decoded by brute force. In homomorphic encryption (33), data are encrypted with a public key and sent to an outside, potentially untrusted source to perform computations. That party never decrypts the data, but instead operates on the data in their encrypted format. The party sends the encrypted results back to the originator, who, with a private key, can decrypt the results.

Encryption and decryption are notoriously slow, so performing large-scale genomic analyses on encrypted data has been prohibitively time-consuming. However, recent work has improved those algorithms through parallelization—a programming technique that divides an application workload into multiple parts, each of which can be run simultaneously on different systems, processors, or cores. In 2018, the winning team of the iDASH Secure Genome Analysis Competition implemented a logistic regression approximation for GWASs that was 30 times faster than the competing SMC solution (8). They did so by parallelizing the execution of matrix operations, efficiently encoding the encrypted data, leveraging approximate arithmetic, and optimizing several cryptographic subroutines. These improvements generalize beyond GWAS computation, enabling homomorphic encryption solutions in other domains that require large-scale statistical analyses on encrypted data. The following year, the winning iDASH team reduced the time necessary to perform imputation on 80,000 SNPs to less than 25 seconds (50).

### Differential Privacy

Differential privacy is a privacy mechanism that adds noise to a database query result such that the entity submitting the query cannot determine whether any particular individual is a member of that database or not. This approach addresses membership inference and reidentification attacks. The more noise the mechanism adds, the less likely it is to infer membership, which provides stronger privacy guarantees; however, stronger privacy guarantees may render the data less useful.

The concept of epsilon-differential privacy mathematically formulates the privacy guarantee through a parameterized epsilon value that defines an inverse privacy budget—the higher the value, the lower the privacy (25). In a study that used differential privacy in a GWAS, Uhlerop et al. (86) advocated for the reasonable release of minor allele frequencies for both cases and controls in a way that does not compromise privacy and permits sharing of chi-squared statistics and *p* values for relevant SNPs.

### Controlled Access

One form of privacy-preserving technology is a suite of services that permit an end user to sign in at a portal (authentication) and access different federated resources depending on the privileges assigned to that user (authorization). Users who apply for access to controlled data resources generally must demonstrate a legitimate research purpose and appropriate qualifications. The controlled access mechanism is how most institutions protect their privacy-sensitive genetic and genomic data. The National Institutes of Health, for example, mandates that all the data from the research it funds be made publicly accessible via controlled access. dbGaP is one such collection of National Institutes of Health data under controlled access (55). One way to implement the controlled access approach is to channel data requests through a data access committee—a group of individuals who serve as key institutional data stewards to evaluate access requests on a case-by-case basis. It is common for a data access compliance office to coordinate the review of data access requests to enable the streamlined and cost-effective administration thereof (48). Cheah & Piasecki (16) suggested that a data access committee should not only protect privacy but also promote data sharing, motivate data producers, and encourage data reuse with transparent, simple, and clear application procedures. Rahimzadeh et al. (65) contended that automated decision support for data access requests improves the auditability, consistency, and efficiency of the data access process and ultimately yields fairer outcomes for the research community. The Data Use Oversight System is the Global Alliance for Genomics and Health (GA4GH) implementation of an automated decision support system for automating the genomic data access process (11).

### Computational Abstractions

There are several computational abstractions that may be leveraged in a federated environment. In this section, we discuss each of these abstractions with respect to privacy and security.

#### Hardware-assisted secure computation.

In most operating systems, there exists a user identity called a privileged user that has access to all the data on the system, including data on disk, in main memory, and in the processor caches. If this identity is compromised, then any privacy-sensitive data on that system are at risk of being compromised as well, which is referred to as a back-door threat. Intel Software Guard Extensions (SGX) was first introduced in 2015 with the aim of providing a trusted execution environment in which applications can protect critical code and data against malicious privileged system code. In SGX, the code is divided into a trusted part (which processes protected data) and an untrusted part (which does not process protected data). Privileged users do not have access to the trusted part of the application when it is running on the SGX processor, thereby eliminating the back-door threat (20). The SGX chip has been leveraged to securely run genomics analyses on systems in order to prevent other applications running on the same system from having access to private data. Two examples include works by Carpov & Tortech (12) and Sadat et al. (69) that leveraged SGX to protect privacy and simultaneously accelerate computational performance in a GWAS.

#### Physical machines, virtual machines, and containers.

A physical machine, also known as a bare-metal machine, is the collection of hardware components (e.g., disk, CPU, and memory) and software components (e.g., kernel and applications) dedicated to and managed by a single operating system. By contrast, a virtual machine is an application that runs on a physical machine’s operating system, abstracting the physical components to allow multiple operating systems to run concurrently on a single physical machine. Containers are lightweight virtual machines that abstract only the elements of an operating system and application stack that must be provided for a given purpose, excluding components of the operating system that are not needed for that purpose. For federation, both virtual machines and container architectures allow for software to be distributed readily and portably among federation members (62). While there are known security risks associated with running certain types of container software that expose privileged user access, these risks are being reduced via newer container architectures (61).

#### Local versus cloud computing.

On-premise (local) computing is a collection of servers, storage devices, networking equipment, power supplies, and so on that operate within the boundaries of an organization. Having dedicated infrastructure in-house entails both the capital expense of purchasing the equipment and the operating expense of managing and running it. Cloud computing is infrastructure that organizations can rent. Cloud consumers still incur the expense of renting time but do not incur any expense to purchase or manage the equipment. Cloud computing is especially beneficial for small organizations that do not have resources to own and run their own computing infrastructure or for any organization that wants to focus its human resources on tasks other than the management of computing infrastructure. Moreover, secure cloud platforms are gaining traction in genomics as a mechanism to share controlled access to data that cannot move for privacy or technical reasons (70, 84, 85).

## FEDERATED COMPUTING TRUST ARCHITECTURES

**Figure 4:**
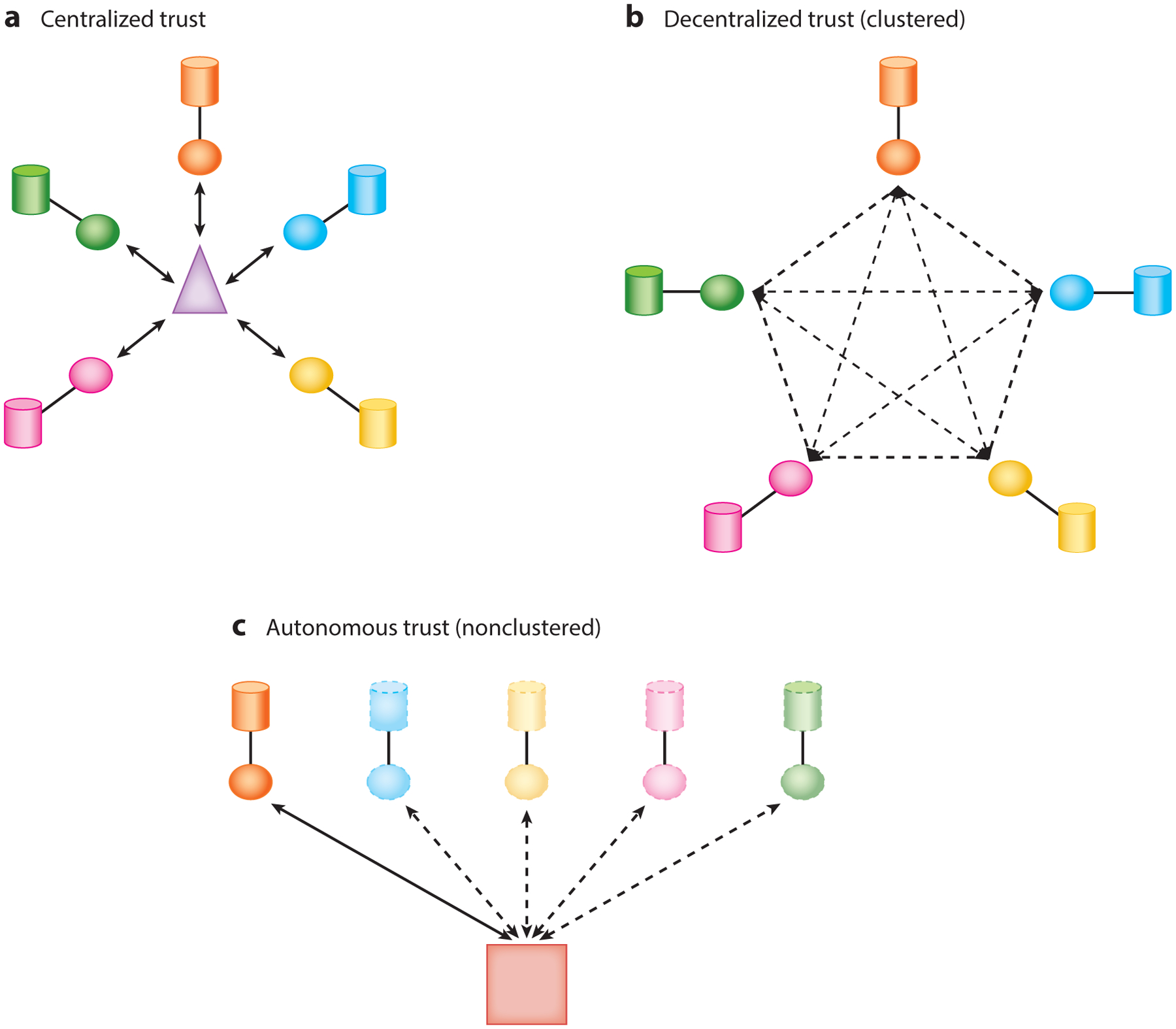
Trust architectures. (*a*) A centralized trust running external to the federation. (*b*) A decentralized trust in which any central coordination is distributed across the cluster. (*c*) A nonclustered environment in which trust is established pairwise between the data consumer and each data owner.

In this review, we consider a federation to be a group of one or more organizations, each with its own privacy-sensitive datasets, that form a single network in which those datasets may be shared in a legally compliant, privacy-preserving manner. Apart from the many technical details that are required to deploy such a solution, at the core of the federation is the trust that participants will comply with the policies set forth to protect the privacy of the individuals who have provided their data and the protocols that enforce the integrity of the federation results. We define three trust architectures of federated computing for genomic data: clustered with centralized trust, clustered with distributed trust, and nonclustered autonomous trust (see Figure 4).

### Centralized Trust

The trusted organization in a centralized trust architecture is an authority that each member of the federation relies upon to establish and maintain overall protocol integrity. In federated learning, for example, updates to the global model are periodically aggregated by the central broker and distributed to each of the federation members. In a controlled access environment, the central authority issues certificates, stores public keys, and provides identity services to authenticate and authorize user access to systems and files (1). One solution leveraging a centralized trust architecture is the Genomics Research and Innovation Network, which consists of a database of phenotypes and genotypes federated over three participating hospitals, harmonizing the institutional research board protocols of each participating hospital (56). This solution requires obtaining the original research participants’ consent to use their data in this broader context and to recon-tact them in order to collect additional data, enroll them in additional studies, or inform them of potentially medically actionable results.

For GDPR compliance, the creation of a centralized trust raises questions regarding the GDPR role of each organization. It is imperative to categorize each of the organizations acting as network nodes as data controllers, joint data controllers, data processors, or nonregulated actors not engaged in the processing of identifiable personal data. For federated analysis, it is optimal to structure a centralized trust as follows. Each node (i.e., organization) that contributes personal data to the federation should be considered a controller of the personal data that it processes. If the identifiable data are processed using third-party hardware or virtual computing resources, then the third parties should be considered the data processors (41). The results that each node contributes to the central analysis node should not contain personal data. Consequently, once the overall federated analysis is synthesized from the organizations acting as network nodes, the final output should not contain personal data.

The result of this structure is that each node processes the personal data at its disposal and must ensure legal compliance for its own personal data-processing activities alone. Because no nodes act as joint controllers, the potential liability of each node is limited to that which results from the analysis of personal data that it processes. Neither the central node nor the recipients of downstream analysis outputs act as data controllers or data processors (27, 29, 95). To achieve this result, each participating node should create a list of the data elements it processes and determine whether these constitute personal data. Each participating node should also create a list of the data elements that it shares with the central analysis node and confirm that none of these data elements constitute personal data. The central node should confirm that the data elements it receives do not constitute personal data, either alone or in combination with one another. The organizations engaged in the federation should ensure that the final outputs of the analysis are not identifiable. Each node should separately ensure that it does not have at its disposal a “means likely reasonably to be used” to perform the reidentification of the concerned individuals, using the available information (9, 10, 64).

Additional measures can be implemented to further ensure that the information that a node shares with and receives from other nodes in the network does not create a risk of reidentification. For example, pre-onboarding trust verification mechanisms can be adopted to ensure that nodes participating in a federation can be presumed trustworthy and will not engage in conduct that could create a risk of individual reidentification, e.g., by ensuring that each node has a bona fide scientific or clinical purpose for engaging in the federation and engages personnel with the necessary technical and scientific training to implement the intended analysis in a manner that reduces the risk of individual reidentification. A second measure is to use contracts that bind the institutions participating in the centralized trust to perform their role in compliance with its policies and to avoid conduct that could lead to individual reidentification (41). A third critical consideration is ensuring that the final federation outputs do not enable the reidentification of the research participants that contributed data to the analysis; one approach is to release the outputs in a registered access or controlled access database, and another is to add noise or perform other modifications to the data to reduce the risk of individual reidentification.

### Decentralized Trust

One criticism of the centralized model is that it concentrates power in a single organization. In an inherently distrustful environment (e.g., a federation among industry competitors), this may preclude an organization from joining the federation. Conversely, in an inherently trustful environment, there is no need to select a central authority. In a decentralized trust architecture, there is no central authority. Trust and agreement among members of the federation are arrived at variously by peer-to-peer majority voting, zero-knowledge proofs, or some other distributed consensus protocol.

#### Swarm learning.

Warnat-Herresthal et al. (93) introduced swarm learning, which achieves decentralized trust by exchanging the role of the central federation authority among the federation members. Swarm learning still uses a central server, but that server is elected among the federation members and changes over the life cycle of model training. It is expected that each federation member will be an aggregation server at some point over the life cycle of the federation. This method leverages a blockchain to manage a distributed ledger and a smart contract for onboarding new federation members, electing the federation authority, and merging model parameters. Warnat-Herresthal et al. (93) used their swarm learning approach to train a classifier on transcriptomic data for predicting disease states in COVID-19, tuberculosis, and leukemia. By decentralizing the federation, swarm learning keeps sensitive data in place; requires no exchange of raw data (encrypted or plaintext); guarantees secure, transparent, and fair onboarding of federation members without a central custodian; allows parameter merging with equal rights for all members; and protects machine learning models from man-in-the-middle attacks.

#### Incremental learning.

Another variation of federated learning that uses decentralized trust is incremental learning (75). This solution entails a classifier of datasets distributed across multiple organizations. Models are trained at one organization using its local data, after which the model parameters are sent to the next organization, which updates the model parameters using its local data. The model is passed through all the organizations participating in the federation and is updated according to their local data. The model may cycle through the organizations for multiple rounds of training until it converges or a specified number of rounds is reached.

From the perspective of data protection law, similar methods of achieving compliance can be recommended for the decentralized trust as for the centralized trust. The centralized trust is preferred when one of the participating organizations is a logical candidate to securely aggregate local analysis results. In instances in which there is no evident custodial node, a decentralized trust might be beneficial. In a decentralized trust, the following distinctions can be relevant in assessing and mitigating the risk of individual reidentification and in assigning responsibilities among network participants.

The federation members should establish a contractual agreement that defines the commitments, organizational measures, and technological precautions that each node must adopt. This can be challenging to achieve if there is no central node that bears formal organizational responsibilities for data custodianship. That is, often there is no central custodial node that is suitable to bear responsibility for ensuring that each other node respects the contractual commitments applicable to the nodes in the network.

To resolve this challenge, each federation member can bind itself to a multilateral contract between that node and all other participating nodes, establishing the responsibilities of each node. This contract can elaborate the categories of organizations and actors that are authorized to act as network nodes and the technical and organizational measures that such nodes must implement to ensure that the information shared between nodes remains nonidentifiable (27, 41). The measures described should be sufficient to safeguard against reidentification attacks, model-poisoning attacks, and other attacks that one or a small number of malicious nodes might attempt to perpetrate through their participation in the federation. Because there is no central custodial node that is responsible for performing the verification of compliance on the part of each federation member, it is a best practice to integrate into the multilateral contract a right for each participating node to compel an audit of another specified node for compliance. Alternatively, each node could be subject to independent third-party audits of their compliance with the specified technical, organizational, and contractual terms at prespecified intervals (27, 41).

### Autonomous Trust

In an autonomous trust environment, there is a distinct trust agreement established between the organizations responsible for the stewardship of identifiable personal data and the downstream organizations that request that analyses be performed on such data. Organizations acting as stewards of identifiable personal data may well be unaware of one another. An example of an autonomous trust federation for processing genomic data comes from Casaletto et al. (13), who developed and shared a container with BioBank Japan to run against a genomic variant dataset from a case–control study of *BRCA1* and *BRCA2* variants. Due to data protection regulations, the data were not allowed to be shared outside the institution. By running a containerized workflow on the data in situ, the authors were able to classify genomic variants that were previously unclassified. In a second example, a team of pediatric cancer researchers from the Treehouse platform shared an RNA sequencing analysis container with partner hospitals that were treating pediatric patients with tumors that had proven difficult to treat (88). While these hospitals were not at liberty to share the actual RNA sequencing data, they were able to share the gene expression calls estimated by the container; through comparative gene expression analysis against a larger cancer cohort, the research team was able to provide useful new insights for approximately 70% of these patients. One approach for safeguarding autonomous trusts, to test that such containers produce the agreed-upon output (and do not leak sensitive data), is for the data steward to create a small test dataset, run the container against it, and examine the output.

The autonomous approach may also be used in situations where the data are not necessarily sensitive but rather are too large to transfer. Keeping the data in place and moving analytic code to the data is the central theme of the big data paradigm. The autonomous approach may further be used in situations where the data controller does not have the means to analyze its own data and thus engages a data processor to perform the analyses.

For purposes of compliance with data protection law, each node acting as a data steward would be considered a data controller, while each third-party service provider that provides computational resources to a data steward node would be a processor. The nodes performing direct analyses should not be construed as controllers, joint controllers, or data processors, since these nodes do not have access to identifiable personal data and do not determine the purposes and means of personal data processing. To ensure this, contractual agreements should be implemented between each producer node and each user node that provides analysis software, establishing that the local nodes act as controllers and that the requesting users’ role is to receive nonidentifiable anonymized data resulting from the local analyses.

The data steward nodes should ensure that none of the outputs shared with analysis recipient nodes constitute identifiable personal data. The data steward nodes should further accept formal responsibility for selecting, implementing, and/or vetting the analyses that the user nodes submit. To ensure that the analysis recipient nodes are not construed as data controllers or data processors, technical mechanisms should be used to limit the queries that the analysis recipient nodes can submit to the data steward nodes. The data steward nodes should have an organizational and/or technical procedure for reviewing and approving the analysis queries that analysis recipient nodes submit prior to their implementation. This helps to ensure that the data steward nodes continue to act as the sole controllers of the concerned personal data, rather than acting as joint controllers in collaboration with the querying analysis recipient nodes (29, 82, 87).

### Hybrid Architectures

Federated solutions for genomic data can mix different trust models in the same architecture; one example of this approach is the Canadian Distributed Infrastructure for Genomics (CanDIG) platform (24). The designers of this federated framework explicitly chose to decentralize authentication and authorization because members of the federation belong to different provinces in Canada. Authentication relies on the identity mechanisms of each of the participating sites, and users log in with their home site credentials rather than with a centralized CanDIG identity. Authorization decisions are made locally at each site, based on the trusted user identity and the nature of the request.

In addition to using the decentralized model for authentication and authorization, CanDIG supports controlled access for registered research users. Controlled access is explicitly granted by data access committees, and researchers with controlled access credentials can access and query datasets. Registered access users sign up and agree to terms of service but have very limited querying ability, and can only query datasets that explicitly allow such access.

## LIMITATIONS TO ADOPTING FEDERATED SOLUTIONS

Common impediments to broader uptake of federated analysis include the absence of data standards or limited adherence to existing standards, and often irreconcilable interpretations of applicable data protection norms.

A significant rate-limiting factor is that there is not yet clear guidance on best practices for regulatory compliance. National regulators and institutions often interpret data protection norms differently. Furthermore, clinical sites and research institutions frequently share data on a voluntary basis to contribute data to the biomedical data commons and foster its productive downstream use; however, the prospect of such contributions entailing legal liability for the data contributors can deter voluntary data sharing. While best practices are starting to emerge (4, 26, 74, 94), there is no current standard of practice, so each individual regulator and regulated party determines its own approach to regulatory compliance. This can lead to fragmented data protection compliance emerging in different jurisdictions and among different institutions that produce, use, or share data. Ultimately, what will drive progress is the development of broadly accepted policy frameworks, promulgated both by regulators and organizations representing regulated parties and civil society (66).

Other impediments to the adoption of federated solutions involve the complexity of designing, implementing, and deploying federated solutions that preserve privacy. In particular, the lack of software development standards and infrastructure deployment best practices for privacy-preserving federated solutions impedes organizations from participating in an interorganizational federation. Federated analysis requires that the data a priori meet quality and formatting standards, given that the method developers cannot directly interact with the actual input data. While the type of input data varies somewhat by the method, most methods require some form of genetic variation data and some form of phenotypic data. Genetic variants are commonly represented in Human Genome Variation Society (HGVS) (23) or Variant Call Format (VCF) (22) nomenclatures, which are well understood but somewhat imprecise. The GA4GH Variant Representation Specification (90) addresses these issues but is not yet widely adopted. For phenotypic or clinical data, the advent of electronic health records has spurred the adoption of the Health Level 7 (HL7) Fast Healthcare Interoperability Resources (FHIR) standard (3, 5) and, more recently, the GA4GH Phenopacket standard (46), but where these standards are not yet adopted, mapping unstructured electronic health records to a structured data standard remains a difficult problem (43). Consequently, there is a limited volume of data that meets data standards, with additional data following ad hoc standards or remaining unstructured. This may be a temporary situation, however; as more software tools emerge that work with data standards, adhering to the standards will ultimately become a cost-saving decision.

## CONCLUSION

The major impediments to sharing genomic data arise more from ambiguous regulatory requirements than from technological limitations. In principle, federated analysis can overcome these impediments to enable data sharing while respecting data privacy or data sovereignty restrictions. While the exact approaches differ, the principle remains consistent: By keeping the sensitive data under the control of the data controller and sharing analysis software to execute on the data controller’s secure system, federated analysis can distill sensitive information down to information that is less sensitive and can therefore be shared more openly while still helping to advance knowledge. Nonetheless, the uptake of federated analysis approaches has been hindered by uncertainties around regulatory compliance, of which the GDPR has been the most noteworthy. Few organizations engaged in complex, consortium-level data-sharing activities have the appetite to bear significant regulatory risk. These risks can prove considerable for organizations that are categorized as data controllers and data processors, as the interpretive ambiguities inherent in data protection law create a potential for unintentional noncompliance. For organizations categorized as joint controllers, the additional prospect of bearing liability for the activities of other collaborating controllers creates heightened compliance risks. These risks can deter data sharing altogether.

In essence, by ensuring that any data egress from the controller’s node consists only of nonidentifiable, nonpersonal data and that any reidentification of these data is highly unlikely, organizations can minimize the practical risks of personal data disclosure and the related risk of data protection law noncompliance. Recent advances in computational data privacy have produced a family of approaches that are robust against many forms of cyber threats, and some even offer a quantifiable level of security; while the computational cost of these methods currently discourages widespread adoption, new hardware developments are making them more tractable. But while technical, organizational, and contractual privacy safeguards can mitigate risk, they cannot eliminate it completely, and the data steward or data controller still bears the largest legal compliance burden. In the future, legislators and regulators must implement both laws and regulatory guidance that diminish both the compliance costs and the prospect of liability for data controllers and data processors that are engaged in prosocial uses of information to facilitate healthcare and research.
